# A Coordinated Translational Control Mediated by eEF2 Phosphorylation Safeguards Erythroid Differentiation

**DOI:** 10.3390/ijms26104801

**Published:** 2025-05-16

**Authors:** Yao Ma, Haozhuo Song, Siming Liu, Wenjing Yu, Guanying Feng, Cuiping Yang, Zhiduo Liu

**Affiliations:** 1 Department of Immunology and Microbiology, Shanghai Institute of Immunology, School of Medicine, Shanghai Jiao Tong University, Shanghai 200025, China; ma_yao_0612@sjtu.edu.cn (Y.M.); songhaozhuo@sjtu.edu.cn (H.S.); simingliu2017@shsmu.edu.cn (S.L.); wenjingyu@shsmu.edu.cn (W.Y.); 2 Shanghai Key Laboratory of Embryo Original Diseases, School of Medicine, Shanghai Jiao Tong University, Shanghai 200030, China; fengguanying@126.com; 3 State Key Laboratory of Systems Medicine for Cancer, School of Medicine, Shanghai Jiao Tong University, Shanghai 200025, China

**Keywords:** eEF2, elongation, protein synthesis, erythroid differentiation, erythropoiesis

## Abstract

Translational control is crucial for maintaining cellular homeostasis, yet the distinct features and regulatory requirements governing protein synthesis during erythropoiesis remain unclear. Here, we reveal that erythroid cells exhibit an extraordinarily high demand for protein synthesis, which is required for their differentiation but also implies the need for tight regulation to prevent excessive erythropoiesis. Notably, we identify significant phosphorylation of eukaryotic elongation factor 2 (eEF2) at threonine 56 during erythroid differentiation, which reduces protein synthesis and acts as a molecular brake to limit unchecked erythropoiesis. This is evidenced by elevated red blood cell counts in peripheral blood and increased incidence of blood hyperviscosity and thrombosis in eEF2_T56M mice, which are deficient in eEF2 phosphorylation. Mechanistic studies demonstrate that eEF2 phosphorylation selectively regulates the translation of a subset of proteins, including NFE2, which partially mediates the effects of eEF2 modification. Collectively, our findings highlight a previously unappreciated role for translational control in achieving efficient and balanced erythropoiesis, with eEF2 phosphorylation serving as a critical protective mechanism against hyperactive erythropoiesis and offering a potential therapeutic target for hematologic disorders such as polycythemia vera.

## 1. Introduction

Erythropoiesis is a tightly regulated process involving hierarchical differentiation stages, in which hematopoietic stem and progenitor cells (HSPCs) stepwise differentiate into lineage-committed erythroid progenitors and precursors, ultimately giving rise to mature circulating red blood cells (RBCs). Approximately 200 billion erythrocytes must be produced each day to ensure efficient oxygen transport, a process achieved by coordinating erythrocyte proliferation, differentiation, and apoptosis [1]. Dysfunction in erythropoiesis can lead to various hematological disorders. Defective erythropoiesis results in diseases like sickle cell anemia, Diamond–Blackfan anemia (DBA), and β-thalassemia [2]. Conversely, excessive RBC production causes polycythemia vera (PV), characterized by increased hematocrit and hemoglobin levels, and is associated with an elevated risk of thrombosis [3]. Therefore, elucidating the molecular mechanisms governing erythropoiesis is crucial for developing therapies to address hematologic disorders.

The stepwise progression of erythroid development is governed by multiple regulators, including cytokines, transcription factors, and chromatin modifiers [4,5]. Growing evidence also reveals the important role of translational control in erythropoiesis. Mutations in ribosomal proteins (RPs) are implicated in the majority of DBA cases [6,7]. The erythroid-specific RNA-binding protein RBM38 interacts with the translation initiation factor eIF4G (eukaryotic initiation factor 4G) to regulate erythropoiesis, and its inhibition impairs erythroid maturation [8]. Moreover, during iron or heme deficiency, activation of heme-regulated eIF2α kinase (HRI) leads to eIF2α phosphorylation, which suppresses globin translation while selectively promoting ATF4 translation to mitigate oxidative stress and prevent ineffective erythropoiesis [9,10]. A recent study also showed that eIF4E modulates erythroid maturation, with its overexpression arresting cells at an early erythroid stage [11]. Despite these insights, how protein synthesis is maintained at appropriate levels during erythroid differentiation under steady state and the potential physiological constraints governing this process remain largely unexplored.

Eukaryotic elongation factor 2 (eEF2) is essential for translation elongation, facilitating tRNA translocation along the ribosome [12]. Its activity is predominantly regulated through phosphorylation at threonine 56 (Thr56), a modification that inactivates eEF2, slows down the elongation rate, and ultimately represses protein synthesis [13]. eEF2 kinase (eEF2K), a calcium/calmodulin (CaM)-dependent enzyme, is the only known kinase in mammals that phosphorylates eEF2 at Thr56, thereby inhibiting its activity and suppressing protein synthesis [14]. While eEF2 phosphorylation has been explored in cancer and neurological disorders [15,16,17], its regulatory role in erythropoiesis remains undefined.

In this study, we show that erythroid cells exhibit an exceptionally high demand for protein synthesis, which far exceeds that of other cell types and is critical for their differentiation. However, negative regulation by eEF2 phosphorylation is also required to maintain protein synthesis at optimal levels. In eEF2_T56M mice, which harbor a mutation abolishing eEF2 phosphorylation, we observed increased RBC counts in peripheral blood, concomitant with a heightened risk of thrombosis and blood stasis, strongly supporting the role of this modification in preventing overactive erythropoiesis. Mechanistically, eEF2 phosphorylation modulates erythroid differentiation in part by selectively regulating NFE2 translation, a transcription factor that controls the expression of key erythroid genes, including β-globin [18]. Overall, our findings highlight eEF2 phosphorylation as a pivotal regulator of erythropoietic homeostasis through coordinated translational control.

## 2. Results

### 2.1. Extraordinarily High Levels of Proteins Are Synthesized During Erythroid Differentiation

**Figure 1:**
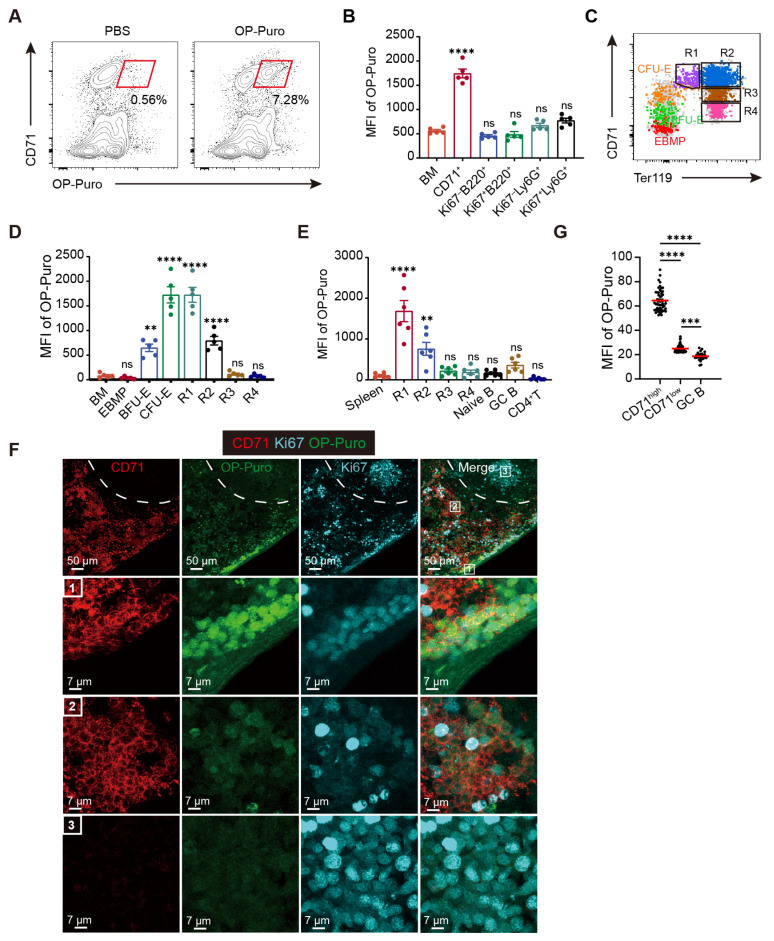
Newly synthesized proteins are significantly increased during erythroid differentiation. (**A**) Representative flow cytometric profiles of global protein synthesis rates in murine bone marrow (BM) cells, assessed 1 h after intraperitoneal injection of O-propargyl-puromycin (OP-Puro) at a dose of 25 mg/kg. (**B**) Protein synthesis rates across distinct BM cell populations were quantified by measuring the mean fluorescence intensity (MFI) of OP-Puro incorporation (*n* = 5). (**C**) Flow cytometric profiles illustrating the distribution of erythroblasts along the continuous erythroid differentiation trajectory from erythroid-basophil-megakaryocyte-biased progenitors (EBMPs) to R4 in BM. Color coding: red, EBMPs; green, erythroid burst-forming units (BFU-Es); orange, erythroid colony-forming units (CFU-Es); purple, R1; blue, R2; brown, R3; pink, R4. (**D**) Protein synthesis rates at various stages of the erythroid differentiation trajectory (*n* = 5). (**E**) Protein synthesis rates across distinct cell populations in the spleen (*n* = 6) from mice immunized with sheep red blood cells (SRBCs). (**F**) Representative confocal images showing the localization of OP-Puro (green) in the mouse spleen 1 h after injection (red: CD71; cyan: Ki67). White dashed lines separate the red and white pulp of the spleen. Numbered boxes represent distinct cell populations with varying OP-Puro expression. Boxes “1” and “2” indicate CD71^+^ erythroblast subsets in the red pulp, and box “3” indicates germinal center cells in the white pulp. Scale bars: 50 µm or 7 µm. (**G**) Quantification of OP-Puro levels in germinal center B cells (CD38^lo^GL7^hi^) and different erythroid subsets in spleen sections (*n* = 50; each point represents an individual cell). Data are presented as mean ± SEM. ** *p* < 0.01, *** *p* < 0.001, **** *p* < 0.0001; ns, not significant. Statistical significance was assessed relative to the “BM” group (**B**,**D**) or the “Spleen” group (**E**).

To evaluate the dynamics of protein synthesis during erythroid differentiation, we used O-propargyl-puromycin (OP-Puro), a puromycin analog that can incorporate into nascent polypeptide chains, to assess protein synthesis capacity by quantifying OP-Puro intensity [19]. One hour after administration, OP-Puro levels in murine bone marrow (BM) were predominantly detected in cells expressing CD71, a transferrin receptor abundantly expressed on erythroblasts (Figure 1A). In contrast to other BM cell types, including rapidly proliferating Ki67^+^ B cells and neutrophils, CD71^+^ erythroid cells exhibited significantly higher levels of protein synthesis (Figure 1B and Figure S1A). This prompted us to examine the translational characteristics at distinct stages of erythroid differentiation. Erythroid-basophil-megakaryocyte-biased progenitors (EBMPs), erythroid burst-forming units (BFU-Es), and colony-forming units (CFU-Es) were identified based on a spectrum of early surface markers (Figure S1B,C) [20]. Furthermore, hierarchical erythroid precursors were classified into R1–R4 subpopulations according to CD71 and Ter119 expression (Figure 1C) [1,21]. Our results revealed that protein synthesis in the less differentiated EBMPs was at baseline levels, comparable to the average of total BM cells. However, it increased markedly in BFU-Es and peaked at the CFU-Es and R1 stages. As erythroid maturation progressed, protein synthesis declined continuously, reaching the lowest levels in R3 and R4 erythrocytes (Figure 1D). In addition to BM, the spleen is an important hematopoietic organ responsible for extramedullary erythropoiesis [22]. Consistent with the BM results, flow cytometric analysis demonstrated that the majority of newly synthesized proteins originated from CD71^+^ erythroid cells in the spleen (Figure S1D). Specifically, compared to different cell types resident in the spleen of sheep red blood cell (SRBC)-immunized mice, including naïve B, germinal center B (GC B), and CD4^+^ T cells (Figure S1E), the R1 subset displayed the highest level of protein synthesis (Figure 1E).

To complement these findings, we applied immunofluorescence staining to the spleen. Ki67 staining in the white pulp identified GC structures enriched with rapidly proliferating B cells [23], where OP-Puro signals were weakly detected (Figure 1F,G). In contrast, the majority of OP-Puro signals existed in the red pulp, coinciding with CD71 expression. The strongest OP-Puro signals originated from CD71^high^ erythroid cells, likely corresponding to R1 or R2 erythroblasts. Additionally, weaker OP-Puro signals concurred with lower CD71 expression. Taken together, these findings demonstrate that extremely high levels of nascent proteins are produced during erythroid differentiation, and this process is dynamically regulated.

### 2.2. Reduced Protein Synthesis Confers Aberrant Erythroid Differentiation

**Figure 2:**
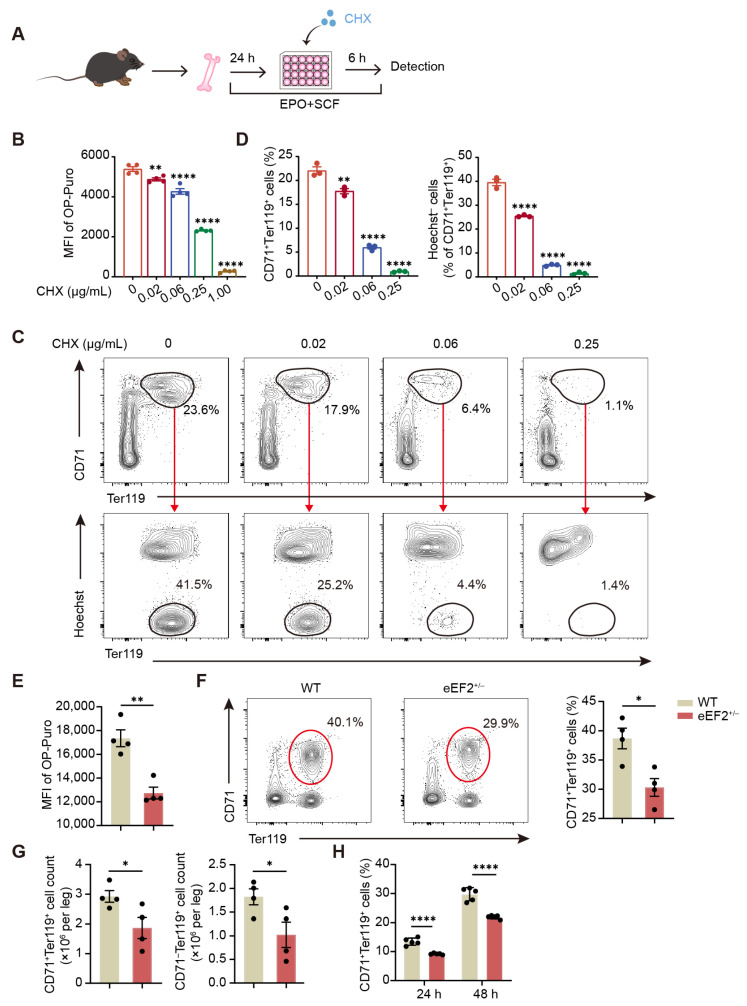
Inhibition of protein synthesis restrains erythroid differentiation. (**A**) Experimental scheme. Isolated BM cells were cultured in vitro for 30 h in a medium containing erythropoietin (EPO) and stem cell factor (SCF) to induce erythroid differentiation, with cycloheximide (CHX) treatment applied during the final 6 h. (**B**) Protein synthesis rates in differentiated CD71^+^Ter119^+^ cells were measured by OP-Puro intensity after CHX treatment as described in (**A**) at the indicated concentrations (*n* = 4). (**C**,**D**) Representative flow cytometric profiles (**C**) and quantification of the percentages (**D**) of CD71^+^Ter119^+^ and enucleated erythroblasts cultured as described in (**A**) at the indicated CHX concentrations (*n* = 3). (**E**) Ex vivo detection of protein synthesis rates in BM CD71^+^Ter119^+^ cells from WT and eEF2^+/−^ mice (*n* = 4). (**F**) Representative flow cytometric profiles (left) and quantification of the percentage (right) of BM CD71^+^Ter119^+^ erythroblasts from WT and eEF2^+/−^ mice (*n* = 4). (**G**) Quantification of the absolute numbers of BM CD71^+^Ter119^+^ and CD71^−^Ter119^+^ cells from WT and eEF2^+/−^ mice (*n* = 4). (**H**) Quantification of the percentage of BM CD71^+^Ter119^+^ cells from WT and eEF2^+/−^ mice following in vitro differentiation for 24 or 48 h (*n* = 5). Data are presented as mean ± SEM. * *p* < 0.05, ** *p* < 0.01, **** *p* < 0.0001. Statistical significance was assessed relative to the control group (CHX: 0 µg/mL) in (**B**,**D**).

To ascertain whether substantial protein synthesis is essential for erythroid differentiation, we established an in vitro erythroid differentiation system in which murine bone marrow cells were induced to differentiate in the presence of erythropoietin (EPO) and stem cell factor (SCF) (Figure S2A). Following in vitro culture, CD71^+^Ter119^+^ erythroblasts emerged, some of which were enucleated, whereas such cells were not detected without cytokine stimulation (Figure S2B). To assess the impact of disrupted protein synthesis on erythroid differentiation, we employed cycloheximide (CHX), a classical inhibitor of protein synthesis that targets translation elongation [24]. CHX was added during the final 6 h of a 30 h in vitro culture with EPO and SCF (Figure 2A). As expected, protein synthesis in erythroblasts was nearly abolished at 1.00 µg/mL CHX, confirming effective translation inhibition (Figure 2B). Despite protein synthesis being reduced by half compared to controls at a lower concentration of 0.25 µg/mL, differentiated CD71^+^Ter119^+^ erythroblasts were virtually undetectable (Figure 2B,C). Intriguingly, even at the lowest CHX concentration tested (0.02 µg/mL), which minimally suppressed protein synthesis, a slight yet statistically significant decrease was observed in the percentages of Ter119^+^ erythroblasts and enucleated cells (Figure 2B–D). Hence, erythroblasts are highly sensitive to fluctuations in protein synthesis, and disturbances in this process interfere with erythroid differentiation.

Furthermore, we investigated the relationship between protein synthesis and erythropoiesis using a genetic approach targeting translational elongation. Eukaryotic elongation factor 2 (eEF2) facilitates elongation by catalyzing tRNA translocation from the aminoacyl (A) site to the peptidyl (P) site and subsequently to the exit (E) site of the ribosome [12]. Using a mouse model with a heterozygous deletion of eEF2 (eEF2^+/−^) (Figure S2C,D), we observed significantly reduced translational activity compared to WT mice (Figure 2E). Remarkably, eEF2^+/−^ BM showed a significant decrease in both the percentage and absolute number of CD71^+^Ter119^+^ erythroid cells (Figure 2F,G), accompanied by reduced CD71^−^Ter119^+^ cell counts, although no detectable differences in erythroid parameters of peripheral blood. Additionally, cultured erythroblasts from eEF2^+/−^ BM exhibited a notable reduction in the percentage of CD71^+^Ter119^+^ erythroid cells compared to WT BM (Figure 2H and Figure S2E), further supporting impaired erythroid differentiation. Together, these data reveal the critical dependence of erythroid cells on optimal protein synthesis rates for the proper progression of differentiation.

### 2.3. eEF2 Phosphorylation Is Tightly Associated with Erythropoiesis

**Figure 3:**
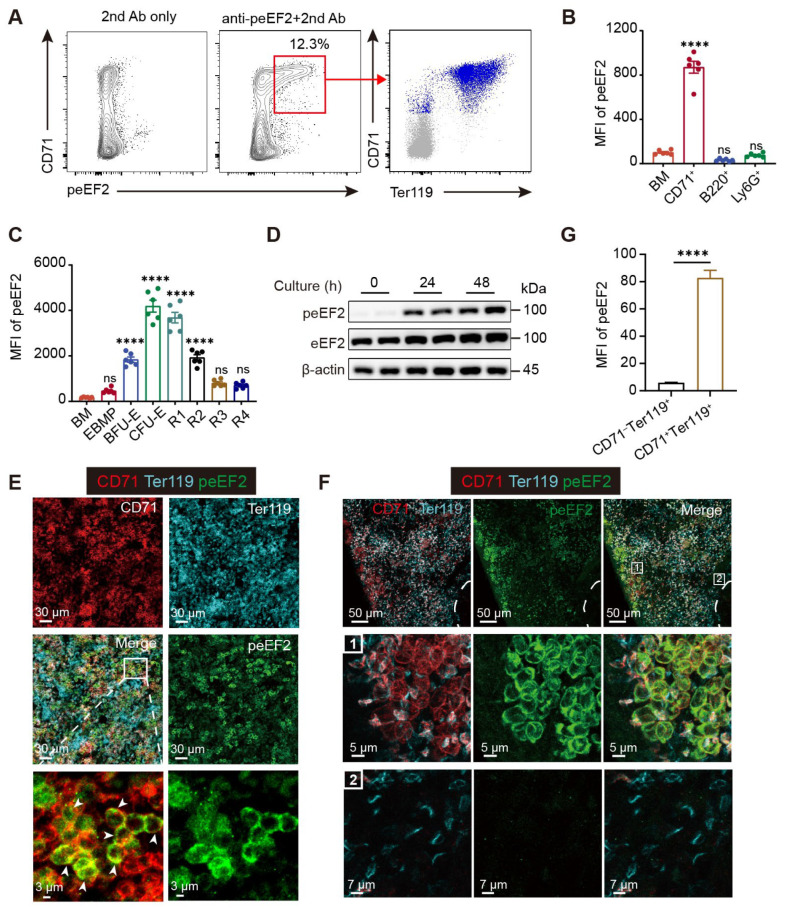
eEF2 phosphorylation is closely interlinked with erythropoiesis. (**A**) Representative flow cytometric profiles of peEF2 expression in BM cells under steady state (left and middle), mapped onto CD71 and Ter119 expression (right). Gray indicates total BM cells, and blue indicates peEF2^+^ erythroid cells. (**B**) MFI of peEF2 levels across the indicated cell types in BM (*n* = 6). (**C**) MFI of peEF2 at various stages of erythroid maturation (*n* = 6). (**D**) Western blot analysis of peEF2 expression in BM cells after in vitro erythroid differentiation at the indicated time points. (**E**) Representative confocal images showing immunofluorescence staining of peEF2 (green) in BM sections from untreated mice (red: CD71; cyan: Ter119). Arrowheads indicate colocalized areas. Scale bars: 30 µm or 3 µm. (**F**) Representative confocal images showing immunofluorescence staining of peEF2 (green) in the spleen from untreated mice (red: CD71; cyan: Ter119). White dashed lines separate the red and white pulp of the spleen. Numbered boxes “1” and “2” indicate CD71^hi^ Ter119^+^ and CD71^−^Ter119^+^ subsets, respectively. Scale bars: 50 µm or 7 µm. (**G**) Quantification of peEF2 levels in the two erythroblast subsets shown in (**F**) on spleen sections (*n* = 50; each point represents an individual cell). Data are presented as mean ± SEM. **** *p* < 0.0001; ns, not significant. Statistical significance was assessed relative to the “BM” group in (**B**,**C**).

Substantial protein synthesis is required for erythroid differentiation, but excessive red blood cell production can lead to disorders like polycythemia vera, suggesting the existence of molecular mechanisms that physiologically limit protein overproduction to prevent uncontrolled erythropoiesis. Through screening for potential regulators of protein synthesis, we observed that eEF2 phosphorylation signals at the T56 site in BM predominantly originated from CD71^+^ erythroid cells under steady state (Figure 3A), significantly exceeding the other BM cell types, including B cells and neutrophils (Figure 3B). Along the erythroid differentiation trajectory, phosphorylated eEF2 (peEF2) signals increased markedly in BFU-Es, peaking in CFU-Es and gradually declining as erythroid cells matured from R1 to R4 subsets (Figure 3C). A two-day in vitro culture system also revealed persistent and prominent eEF2 phosphorylation in differentiating erythroblasts (Figure 3D).

Immunofluorescence staining showed widespread peEF2 signals in BM under steady state, extensively colocalizing with CD71^+^ cells (Figure 3E). Notably, strong peEF2 expression was observed in the red pulp of the murine spleen, where erythrocytes reside. A strong correlation was observed between peEF2 levels and CD71 expression, with the most intense signals in the CD71^high^ subset localized in the peripheral region of the red pulp (Figure 3F,G). Furthermore, under stress erythropoiesis induced by phenylhydrazine (PHZ) [25], we also observed overt peEF2 expression in the spleen (Figure S3A). Apart from BM and spleen, no obvious peEF2 signals were detected in the other tissues we tested, including lymph nodes, liver, and kidney (Figure S3B–D), suggesting that strong eEF2 phosphorylation occurs primarily in erythropoietic organs under steady state. The specificity of the peEF2 antibody was validated by robust peEF2 signals in WT HeLa cells subjected to glucose starvation (GS) (Figure S3E,F), which were absent in HeLa cells carrying a mutation at the T56 phosphorylation site of eEF2. Collectively, these findings indicate that eEF2 phosphorylation is closely associated with erythropoiesis and may play a regulatory role in this process.

### 2.4. The Lack of eEF2 Phosphorylation Markedly Accelerates Erythroid Maturation

**Figure 4:**
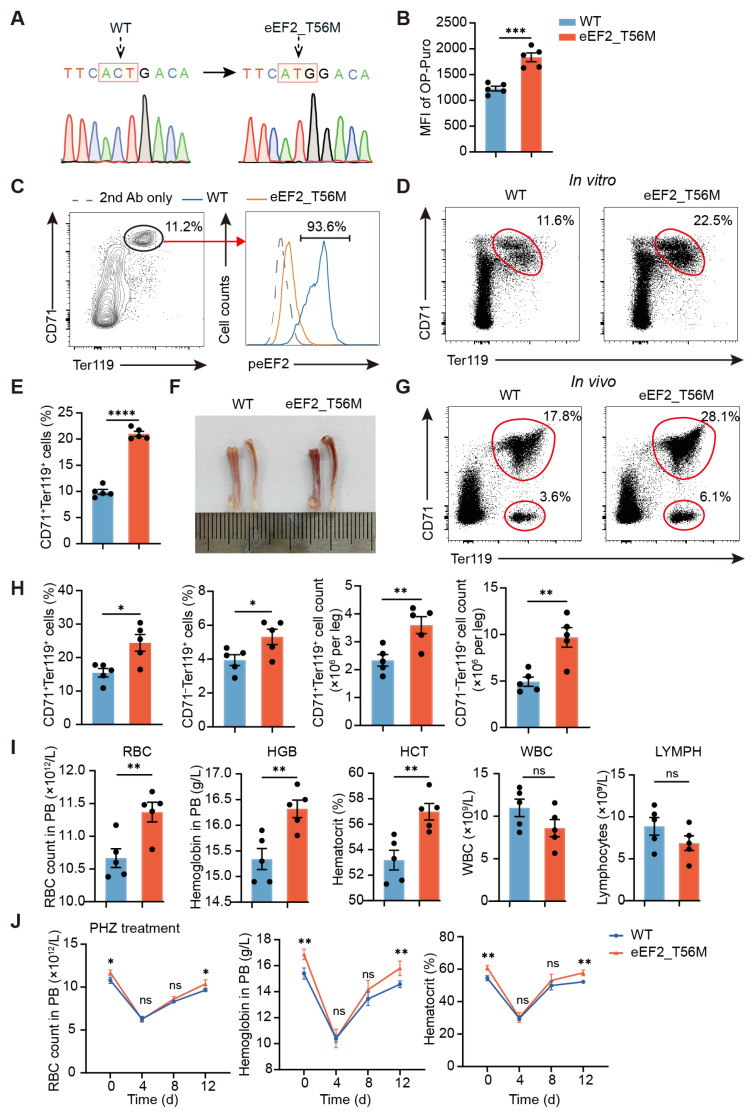
eEF2 phosphorylation negatively modulates erythroid maturation. (**A**) Schematic representation of the eEF2_T56M mouse model, where the 56th amino acid of eEF2 was mutated from threonine (T) to methionine (M). (**B**) Ex vivo detection of protein synthesis rates in BM CD71^+^Ter119^+^ cells from WT and T56M mice (*n* = 5). (**C**) Flow cytometric analysis of peEF2 expression in BM CD71^+^Ter119^+^ erythroblasts after 36 h of in vitro differentiation. (**D**,**E**) Representative flow cytometric profiles (**D**) and quantification of the percentage (**E**) of CD71^+^ Ter119^+^ erythroblasts from WT and T56M BM after 36 h of in vitro differentiation (*n* = 5). (**F**) Representative image of BM from WT and T56M mice. (**G**) Representative flow cytometric profiles of BM CD71^+^Ter119^+^ and CD71^−^Ter119^+^ erythroblasts from WT and T56M mice. (**H**) Percentages and absolute numbers of the indicated erythroid populations shown in (**G**) (*n* = 5). (**I**) Analysis of red blood cell (RBC) counts, hemoglobin (HGB), hematocrit (HCT), white blood cell (WBC) counts, and lymphocyte (LYMPH) counts in peripheral blood (PB) of WT and T56M mice (*n* = 5). (**J**) PHZ-induced anemia and recovery in WT and T56M mice. Analysis of RBC counts, HGB, and HCT at the indicated time points (*n* = 4). Data are presented as mean ± SEM. * *p* < 0.05, ** *p* < 0.01, *** *p* < 0.001, **** *p* < 0.0001; ns, not significant.

To elucidate the role of eEF2 phosphorylation in erythropoiesis, we generated an eEF2_T56M knockin mouse model (hereafter referred to as T56M), where the 56th amino acid of eEF2 was mutated from threonine (ACT) to methionine (ATG) (Figure 4A). This mutation abolishes eEF2 phosphorylation at this site, leading to enhanced translational activity, as indicated by more OP-Puro signals in T56M-derived BM erythroid cells compared to WT controls (Figure 4B). We then evaluated the functional significance of eEF2 phosphorylation in an in vitro erythroid differentiation assay. After 36 h of culture, eEF2 phosphorylation was detected in nearly all CD71^+^Ter119^+^ cells from WT mice, but was absent in T56M-derived erythroblasts (Figure 4C and Figure S4A). Remarkably, the percentage of cultured CD71^+^Ter119^+^ cells from T56M mice was nearly twice that of the control group (Figure 4D,E). Consistent with enhanced erythroid maturation in vitro, T56M BM displayed a deeper reddish hue (Figure 4F), reflecting increased erythroid cell content. Indeed, flow cytometric analysis confirmed a significant increase in the percentage and number of both CD71^+^Ter119^+^ and CD71^−^Ter119^+^ erythroid populations (Figure 4G,H).

Peripheral blood analysis further revealed that T56M mice exhibited higher red blood cell (RBC) counts, hemoglobin (HGB) levels, and hematocrit (HCT) compared to WT mice, with no significant changes in white blood cell (WBC) and lymphocyte (LYMPH) counts (Figure 4I). Meanwhile, the proportions of other major immune cells in BM and spleen were comparable between the two groups (Figure S4B,C), suggesting that the effects of eEF2 phosphorylation are largely restricted to the erythroid lineage. Additionally, in the context of stress erythropoiesis induced by PHZ, T56M mice exhibited accelerated RBC recovery relative to WT mice (Figure 4J). Thus, the T56M mutation promotes erythroid maturation under both steady state and stress erythropoiesis, indicating that eEF2 phosphorylation plays a restraining role to prevent excessive RBC production.

### 2.5. eEF2_T56M Mice Exhibit Increased Susceptibility to Blood Stasis and Thrombosis

**Figure 5:**
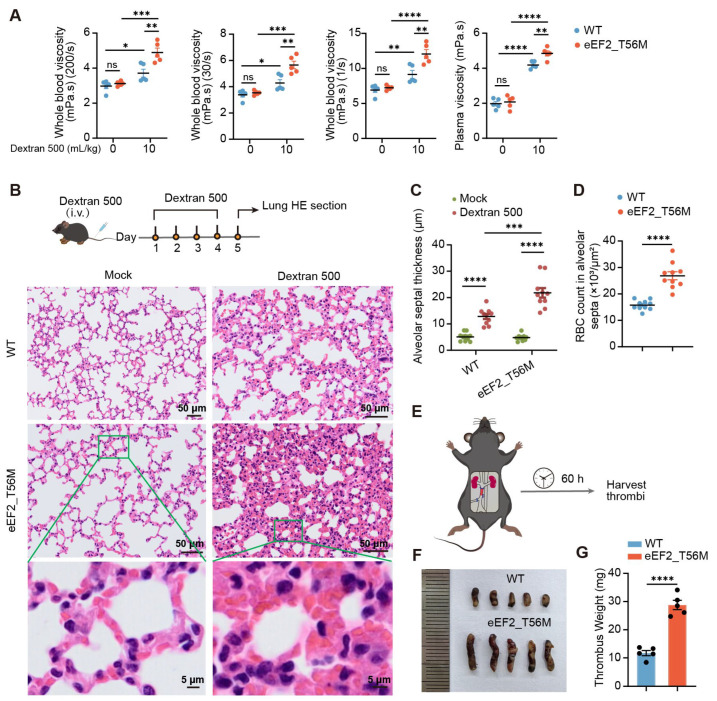
eEF2_T56M mice are vulnerable to blood stasis and thrombosis. (**A**) Quantification of whole blood viscosity (WBV) at varying shear rates and plasma viscosity (*n* = 5). (**B**) Hematoxylin and eosin (HE) staining of lung sections from WT and T56M mice following intravenous injection of dextran 500 (7.5 mL/kg) for four consecutive days. (**C**) Quantification of alveolar septal thickness in WT and T56M mice treated as in (**B**) (*n* = 10 fields of view from 4 lung sections per mouse). Scale bars: 50 µm or 5 µm. (**D**) Quantification of RBC counts within the average area of alveolar septa, as shown in (**B**) (*n* = 10 fields of view from 4 lung sections). (**E**) Schematic representation of the inferior vena cava (IVC) stenosis model. (**F**) Representative image of the thrombus formed in the IVC. (**G**) Quantification of thrombus weight from WT and T56M mice 60 h after IVC surgery (*n* = 5). Data are presented as mean ± SEM. * *p* < 0.05, ** *p* < 0.01, *** *p* < 0.001, **** *p* < 0.0001; ns, not significant.

In light of the link between elevated hematocrit and increased blood viscosity [26], we investigated the alterations in blood rheology. While no significant difference in blood viscosity was observed between WT and T56M mice under steady state, following intravenous injection of dextran 500 for 1 h, a compound known to increase blood viscosity [27], T56M mice exhibited significantly higher blood and plasma viscosity than WT controls (Figure 5A). Prolonged dextran 500 administration for four consecutive days resulted in thicker alveolar septa in T56M mice (Figure 5B,C), accompanied by increased RBC aggregation (Figure 5D). These findings indicate that T56M mice are more prone to blood hyperviscosity and RBC aggregation.

Since RBC aggregation in blood vessels contributes to thrombus [28], a risk frequently associated with PV patients [3,29], we determined whether T56M mice are more susceptible to thrombosis. We employed a mouse model of deep vein thrombosis (DVT) induced by partial ligation of the inferior vena cava (IVC) and complete ligation of the side branches (Figure 5E) [30]. At 60 h post-surgery, the thrombi formed in T56M mice were significantly longer and heavier compared to those in WT mice (Figure 5F,G). Altogether, these findings provide compelling evidence that eEF2 phosphorylation functions as a protective mechanism to maintain erythropoietic homeostasis, the absence of which would result in the heightened incidence of blood rheological disorders in T56M mice.

### 2.6. eEF2 Phosphorylation Depends on NFE2 in the Regulation of Erythroid Differentiation

**Figure 6:**
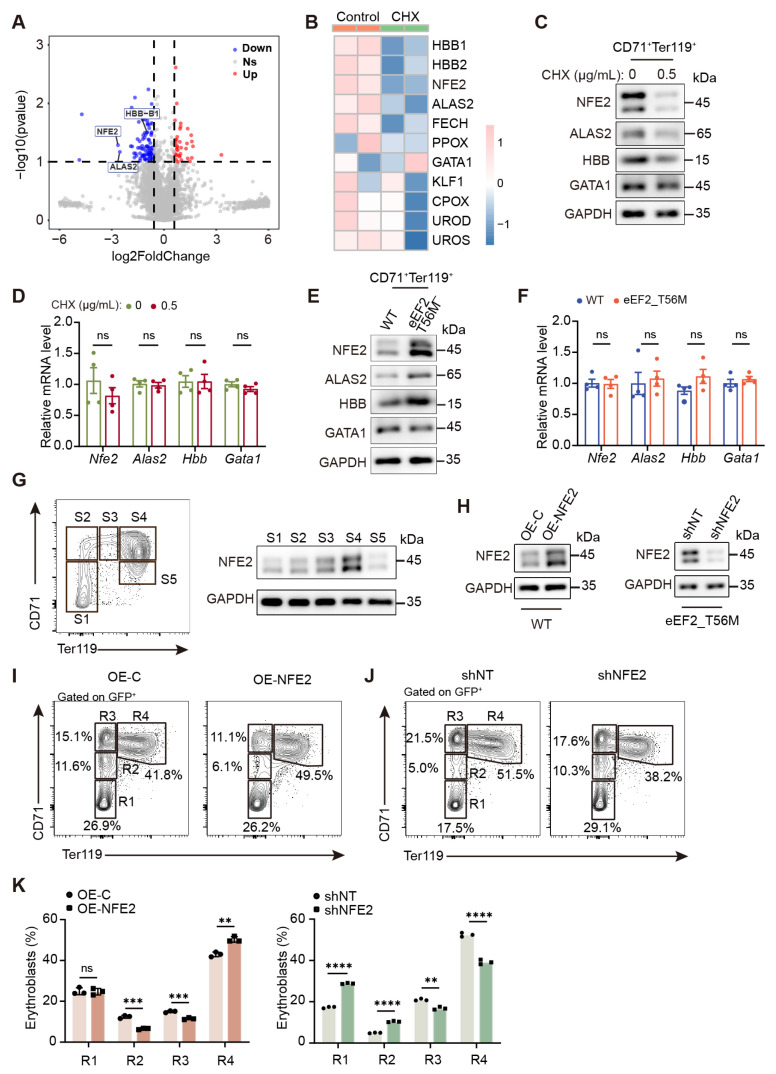
Identification of NFE2 as a potential target of peEF2 in mediating erythroid differentiation. (**A**) Volcano plot showing differentially expressed proteins in sorted CD71^+^Ter119^+^ erythroblasts treated with or without CHX (0.5 µg/mL) during the final 6 h of a 30 h in vitro culture in EPO-supplemented medium compared to untreated controls (fold change > 1.5, *p* value < 0.1). (**B**) Heatmap analysis displaying proteins associated with erythroid differentiation. (**C**) Western blot analysis of sorted CD71^+^Ter119^+^ erythroblasts treated with or without CHX during in vitro differentiation as in (**A**). (**D**) mRNA levels of genes in erythrocytes from (**C**) were determined by quantitative PCR (qPCR) assay (*n* = 4). (**E**) Western blot analysis of sorted CD71^+^Ter119^+^ erythroblasts following in vitro differentiation from BM of WT and T56M mice. (**F**) mRNA levels of genes in erythrocytes from (**E**) were determined by qPCR assay (*n* = 4). (**G**) Gating strategy for S1–S5 erythroblasts of E14.5 mouse fetal liver cells (FLCs) based on CD71 and Ter119 expression (left), and western blot analysis of NFE2 expression at the indicated phases (right). (**H**) Western blot analysis of NFE2 overexpression in WT FLCs (left) or knockdown in T56M FLCs (right). FLCs were transduced with retroviruses encoding NFE2 or NFE2 shRNA, cultured in SCF medium for 12 h, and then switched to EPO medium for an additional 24 h. (**I**,**J**) Representative flow cytometric profiles of R1–R4 erythroid populations in FLCs with NFE2 overexpression (**I**) or NFE2 knockdown (**J**) after 48 h of in vitro culture in EPO medium. (**K**) Quantification of the percentages of the indicated populations from (**I**) and (**J**) (*n* = 3). Data are presented as mean ± SEM. ** *p* < 0.01, *** *p* < 0.001, **** *p* < 0.0001; ns, not significant.

To investigate how translational control influences erythropoiesis, we performed proteomic analysis on sorted BM CD71^+^Ter119^+^ erythroblasts treated with or without CHX during the last 6 h of a 30 h in vitro culture in EPO-containing medium. Differential expression analysis identified a range of downregulated proteins associated with erythroid differentiation following CHX treatment, including the transcription factor NFE2, β-globin (HBB), and essential heme biosynthesis enzymes (ALAS2, FECH, PPOX, etc.) (Figure 6A,B). Western blot analysis confirmed reduced protein levels of NFE2, HBB, and ALAS2 upon CHX treatment, whereas the expression of the master erythroid transcription factor GATA1 remained unchanged (Figure 6C). Intriguingly, quantitative PCR (qPCR) analysis revealed that the mRNA levels of *Nfe2*, *Alas2*, *Hbb*, and *Gata1* were unaffected (Figure 6D). Notably, the proteins downregulated by CHX treatment were significantly upregulated in T56M erythroblasts (Figure 6E), without corresponding changes in their mRNA levels (Figure 6F), suggesting a regulatory mechanism by which eEF2 phosphorylation specifically modulates these proteins at the translational level.

Among these, NFE2 (nuclear factor erythroid 2) exhibited one of the most pronounced alterations. As a transcription factor, NFE2 collaborates with GATA1 and KLF1 to activate β-globin expression [31] and controls the transcription of heme biosynthesis enzymes [32]. Overexpression of NFE2 has been implicated in PV patients [33,34]. These findings collectively point to the critical role of NFE2 in erythropoiesis, leading us to hypothesize that NFE2 is a downstream candidate of eEF2 phosphorylation in erythroid development.

To explore the relationship between eEF2 phosphorylation and NFE2, we employed an in vitro mouse fetal liver cell (FLC) culture system, a highly efficient platform for gene manipulation via viral infection. E14.5 mouse FLCs were sorted into S1–S5 subsets (Figure 6G, left), and robust eEF2 phosphorylation was detected in the S4 subset during in vivo embryonic hematopoiesis (Figure S5A) and in the CD71^+^Ter119^+^ population under in vitro differentiation (Figure S5B). Consistently, in vitro differentiating T56M FLCs showed a higher proportion of CD71^high^Ter119^+^ erythroblasts than WT (Figure S5C). NFE2 expression increased modestly from S1 to S3, peaked in the S4 subset (primarily CD71^high^Ter119^+^), and subsequently declined in S5 erythroblasts (Figure 6G, right), indicating tight regulation of NFE2 expression throughout erythropoiesis.

Notably, NFE2 overexpression significantly accelerated terminal erythroid maturation, characterized by reduced early-stage erythroblasts and increased mature R4 (CD71^+^Ter119^+^) cells during in vitro differentiation (Figure 6H,I,K), mirroring the accelerated maturation observed in T56M mice (Figure 4). Conversely, short hairpin RNA-mediated NFE2 knockdown (shNFE2) in T56M erythroblasts markedly delayed terminal erythroid maturation (Figure 6H,J,K). Overall, these findings reveal a novel translational regulatory axis in which eEF2 phosphorylation governs erythroid differentiation via NFE2, thereby fine-tuning erythropoiesis.

### 2.7. eEF2 Phosphorylation Guides Translational Specificity to Manipulate Erythropoiesis

**Figure 7:**
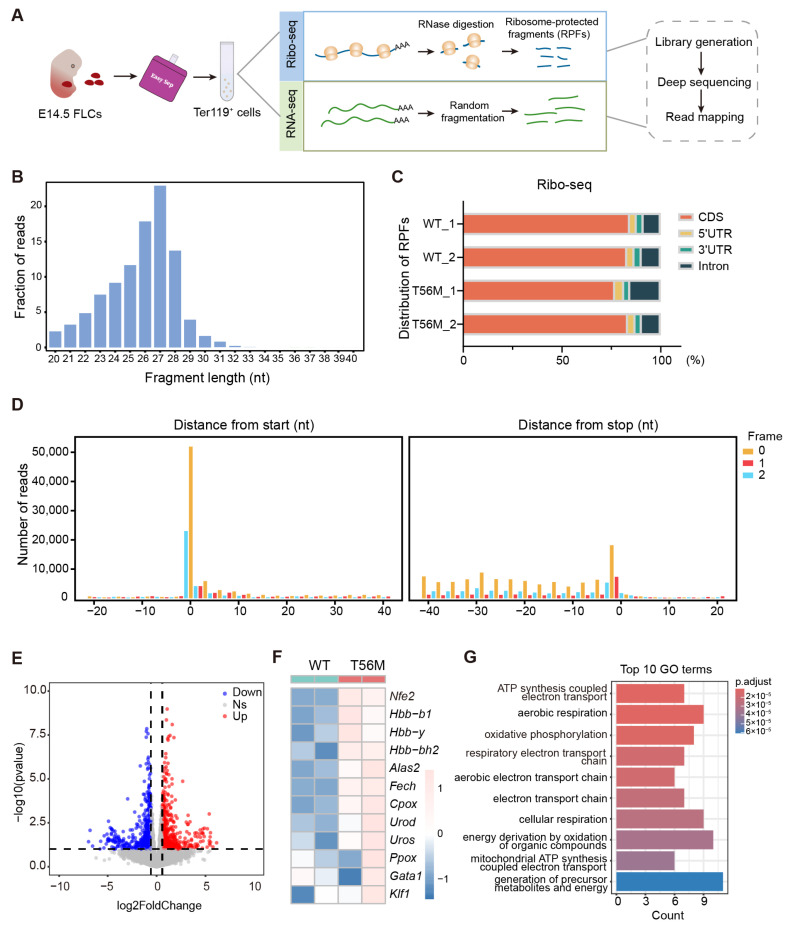
eEF2 phosphorylation orchestrates a unique translational program to regulate erythropoiesis. (**A**) Overview of experimental design. WT and T56M E14.5 FLCs were enriched for Ter119^+^ erythroid cells and then subjected to parallel RNA-seq and Ribo-seq. (**B**) Length distribution of ribosome-protected fragments (RPFs) in Ribo-seq libraries. (**C**) Relative fraction of RPFs mapped to the CDS, 5′ UTR, and 3′ UTR of annotated transcripts. (**D**) Fractions of reads assigned to each nucleotide around the start and stop codons. (**E**) Volcano plot showing distinct gene expression profiles between WT and T56M groups. (**F**) Heatmap analysis of erythroid-related genes in WT and T56M groups. (**G**) GO analysis of transcripts with upregulated translation efficiency (fold change > 1.5, FDR < 0.05).

The foregoing findings prompted us to dissect the translational programs governed by eEF2 phosphorylation. To this end, we performed parallel RNA and ribosome profiling (Ribo-seq) on Ter119^+^ erythroblasts from WT and T56M fetal livers to comprehensively analyze the translational regulatory networks during murine hematopoiesis (Figure 7A). The ribosome-protected fragments (RPFs) we obtained displayed the expected size distribution (25–28 nt) (Figure 7B), coding sequence (CDS) enrichment (Figure 7C), and excellent triplet periodicity (Figure 7D), confirming the high quality of these RPFs.

Integrative analysis of RNA-seq and Ribo-seq identified 588 mRNAs with significantly upregulated translation efficiency (TE, the ratio of the abundances of ribosome footprints and available mRNA fragments [35]) in T56M cells (Figure 7E). Notably, a series of genes with increased TE were associated with erythroid development, including the transcription factor *Nfe2*, heme biosynthesis enzymes (e.g., *Alas2*, *Fech*, *Cpox*, *Urod*), and hemoglobin (*Hbb*). In contrast, the TE of key erythroid transcription factors *Gata1* and *Klf1* remained unchanged in T56M cells (Figure 7F). This observation aligns closely with our previous proteomic data and underscores the pivotal role of peEF2 in directing the translational specificity of *Nfe2*, independent of direct binding or spatial co-localization with NFE2 (Figure S6). Gene Ontology (GO) analysis revealed that mRNAs with significantly upregulated TE were strongly associated with mitochondrial biogenesis, including ATP biosynthesis, electron transport chain, and oxidative phosphorylation (Figure 7G). These pathways are essential for heme and iron–sulfur cluster biosynthesis, which are critical for proper erythropoiesis [36]. Our findings are supported by previous studies demonstrating that the translation of mitochondria-related mRNAs is markedly increased during erythropoiesis, and that mitochondrial dysfunction impairs this process [37]. Taken together, these findings indicate that peEF2 orchestrates a translational program that selectively targets a subset of erythroid genes and mitochondrial function-related genes.

## 3. Discussion

In this study, we revealed the distinct characteristics of protein synthesis during erythroid differentiation and identified a critical protective mechanism mediated by eEF2 phosphorylation, which serves as a molecular brake to ensure optimal and balanced protein synthesis, thereby preserving erythropoietic homeostasis.

Several studies [11,38], including our own, have shown that protein synthesis levels in erythroid cells initially increase and subsequently decline throughout erythropoiesis. This trend aligns with the developmental trajectory of erythroid cells, which begins with an early differentiation phase characterized by rapid proliferation and transitions into a maturation phase marked by reduced proliferative capacity and cell size, ultimately yielding functional red blood cells devoid of most organelles. Strikingly, we observed that protein synthesis rates in early erythroid precursors are extraordinarily high, far surpassing not only those in rapidly proliferating neutrophil and B-cell precursors in bone marrow, but also those in activated germinal center B cells in peripheral lymphoid organs, which are generally regarded as possessing robust protein synthesis capacity for antibody production. This raises an intriguing question about the specific proteins synthesized at this stage, with proteomic analysis providing preliminary insights. Studies from Xu’s and Blanc’s laboratories have revealed that [37,39], in addition to central erythroid transcription factors such as GATA1 and KLF1, proteins with increased abundance during the early phase of human erythropoiesis are predominantly enriched in two categories, with one associated with hemoglobin and heme biosynthesis, which are hallmarks of erythroid differentiation, and the other related to mitochondrial biogenesis, which is consistent with the markedly elevated mitochondrial biomass and potential observed in ProEs compared to HSPCs. While changes in protein stability may contribute, it is likely that the increased abundance of those specific proteins is primarily attributed to the significantly heightened protein synthesis capacity, a hypothesis that requires further investigation with more precise approaches. Another distinct feature of translational control in erythroid cells is their extreme sensitivity to fluctuations in protein synthesis levels, as we found that even minor disruptions can lead to defective erythroid output in vitro. Supporting this, dysregulated erythropoiesis was observed in eEF2_T56M mice, whereas the development of other cell types, such as lymphocytes, remained unaffected. This hypersensitivity may also explain why mutations in over 20 RP genes are implicated in DBA [7]. Together, these two unique characteristics underscore the importance of translational regulation in erythroid differentiation and offer promising therapeutic avenues for hematologic disorders.

Translation initiation is widely considered the rate-limiting step in protein synthesis, and recent research on the regulation of protein synthesis during erythropoiesis has predominantly focused on initiation factors within the translation machinery, such as eIF2α, eIF4E, and eIF5A [9,11,40]. In contrast, our study uncovers a previously unexplored connection between translation elongation and erythroid differentiation, mediated by eEF2 phosphorylation at Thr56. Although the molecular basis underlying its effect on eEF2 function has been extensively studied, its physiological significance remains poorly understood and is largely confined to roles in the brain and skeletal muscle. Remarkably, we observed strong eEF2 phosphorylation signals in erythroid precursors in both bone marrow and fetal liver under steady state, as well as in the spleen during stress erythropoiesis. These findings, along with data from the eEF2_T56M mouse model we developed, establish erythropoiesis as a critical physiological context for understanding eEF2-mediated translational control in vivo.

It may appear paradoxical that eEF2-mediated translational suppression occurs in erythroid cells, which concurrently require extraordinarily high levels of protein synthesis for differentiation. However, such seemingly contradictory regulatory mechanisms are often indispensable for enabling a system to respond rapidly to external stimuli while maintaining homeostasis, as observed in various contexts. For instance, bacterial infections can trigger robust cytokine production by immune cells to combat the invasion, while host-derived negative regulators are simultaneously upregulated to suppress excessive inflammatory responses and prevent tissue damage. Similarly, despite the high protein synthesis demand for effective erythropoiesis, mechanisms such as eEF2 phosphorylation likely act as safeguards to modulate this capacity and ensure optimal erythroid differentiation. The factors responsible for inducing eEF2 phosphorylation in this process remain unclear. eEF2K is currently the only recognized kinase for eEF2 phosphorylation in mammals, yet our data indicate that eEF2 phosphorylation occurs independently of eEF2K, as pharmacological inhibition of eEF2K had no significant effect on peEF2 levels or erythroid differentiation (Figure S7A). Further validation using eEF2K knockout models is warranted to confirm these findings. Notably, the EPO-EPOR axis activates the PI3K/AKT signaling pathway, which is critical for erythroid cell survival, proliferation, and differentiation via serine/threonine phosphorylation cascades [4,41]. Intriguingly, treatment of cultured bone marrow cells with Afuresertib (GSK2110183), an AKT inhibitor, resulted in a marked reduction in eEF2 phosphorylation levels (Figure S7B). This observation suggests that the kinases involved in the AKT signaling pathway may be potential regulators of eEF2 phosphorylation. Although the precise kinases targeting eEF2 remain unidentified, our findings highlight the significance of an eEF2-dependent negative feedback mechanism in ensuring well-regulated erythropoiesis.

In contrast to the notion that impaired elongation universally reduces overall protein synthesis, our results reveal a specific translational control mediated by eEF2 phosphorylation during erythropoiesis, supported by proteomic profiles obtained under CHX treatment, a widely used translation elongation inhibitor targeting eEF2. Among transcripts with upregulated translation efficiency in T56M erythroblasts, a subset of genes directly involved in heme and globin biosynthesis stood out, including *Alas2*, *Fech*, and *Nfe2*. Even subtle changes in these processes can significantly impact erythroid progression, as evidenced by reduced erythroid maturation upon NFE2 suppression in T56M cells, consistent with a previous report linking elevated NFE2 levels to polycythemia vera [33,34]. Another major finding is that eEF2 phosphorylation also guides the translation of mitochondrial proteins. The critical role of mitochondrial biosynthesis in erythropoiesis is underscored by evidence showing that disruptions in mitochondrial protein synthesis, such as those caused by impaired eIF5A function or depletion of mitochondrial transcription factor A (TFAM), severely hinder erythroid differentiation [37,40]. Overall, our studies emphasize eEF2 phosphorylation as an additional regulatory layer beyond gene transcription, adeptly coordinating erythropoiesis through a unique translational program.

It is noteworthy that, despite a concerted effort to drive basal protein synthesis, individual components of the translation machinery exhibit specialized roles in regulating distinct translational profiles. For example, unlike eEF2, eIF4E directs the selective translation of proteins implicated in early hematopoiesis, such as PTPN6 and IGF2BP1, contributing to the maintenance of the early precursor state of erythroid cells [11]. While eIF4E preferentially translates mRNAs with conserved cytosine-rich motifs in their 5′ UTRs [11,42], the mechanism by which eEF2 phosphorylation confers translational specificity remains unclear. Although multiple possibilities may exist, it is tempting to hypothesize that the phosphorylation of a portion of eEF2 molecules reduces active eEF2 availability, making well-translated transcripts with abundant ribosomes more susceptible to ribosome collision and translation arrest. This assumption aligns with the proposed role of eEF2 phosphorylation as a fine-tuning mechanism for regulating massive protein synthesis during erythropoiesis. Future studies are necessary to elucidate the precise mechanisms governing translational selectivity mediated by eEF2 phosphorylation.

Polycythemia vera (PV) is a myeloproliferative neoplasm characterized by elevated hemoglobin and hematocrit levels, most commonly driven by constitutive activation of the JAK2 signaling pathway [3]. Notably, in our study, T56M mutant mice exhibit increased hemoglobin and hematocrit compared to WT mice (Figure 4), recapitulating a hallmark clinical feature of PV. Moreover, T56M mice display an enhanced propensity for thrombosis (Figure 5), a major complication in PV patients. NFE2, a transcription factor upregulated in PV [33,34], is also significantly elevated in T56M mice (Figure 6). These parallels suggest that impaired phosphorylation of eEF2 may play a previously underappreciated role in promoting erythrocytosis, independent of JAK2 mutations. Our results raise the possibility that mutations affecting eEF2 phosphorylation sites, particularly at T56, may exist in a subset of PV patients, especially those lacking the canonical JAK2V617F mutations. Screening for such mutations could help identify individuals at risk, and targeting the translational elongation machinery may represent an innovative and promising therapeutic strategy for this specific patient cohort.

Despite these findings, our study has several limitations. The precise molecular mechanisms by which eEF2 phosphorylation selectively modulates mRNA translation remain to be fully elucidated. Furthermore, identifying the upstream effectors responsible for eEF2 phosphorylation will facilitate the development of targeted strategies to regulate erythropoiesis via translational control.

In conclusion, our study highlights the importance of elaborate translational control during erythropoiesis, revealing eEF2 as a critical regulator of coordinated erythroid differentiation (Figure S8). These findings establish a foundation for developing innovative therapies for hematologic disorders.

## 4. Materials and Methods

### 4.1. Mice

All mice used in this research were on the C57BL/6J genetic background. C57BL/6 mice were purchased from Shanghai Slaccas Company (Shanghai, China). eEF2^+/−^ mice (strain #T027339) and eEF2_T56M mice (strain #T053825) were purchased from Jiangsu GemPharmatech Co., Ltd (Nanjing, China). eEF2_T56M homozygous mice exhibit normal body size, weight, and activity levels under standard housing conditions. For PHZ treatment, mice were intraperitoneally injected with PHZ (50 mg/kg body weight; Sigma, Burlington, MA, USA) once to induce hemolytic anemia. All mice were maintained with standard 12 h light/dark cycles under specific pathogen-free (SPF) conditions. Mice aged 8 to 16 weeks were used for experiments. All animal experiments were approved by the Institutional Animal Care and Use Committee (IACUC) of Shanghai Jiao Tong University School of Medicine.

### 4.2. Cell Isolation and Culture

For bone marrow preparation, femurs and tibias were collected and placed in phosphate-buffered saline (PBS) (BasalMedia, Shanghai, China) containing 2% fetal bovine serum (FBS) (ExCell Bio, Suzhou, China). Bone marrow cells were flushed out using a 2 mL sterile syringe fitted with a 26-gauge needle and passed through a 70 µm cell strainer. After red blood cell lysis with ACK buffer, 1 × 10^6^ bone marrow cells were seeded in a 48-well plate and cultured in Iscove’s Modified Dulbecco’s Medium (IMDM) supplemented with 10% FBS, 1% penicillin/streptomycin, 200 μg/mL holo-transferrin (Beyotime, Shanghai, China), 10 μg/mL recombinant human insulin (Beyotime), 50 ng/mL stem cell factor (SCF) (PeproTech, Cranbury, NJ, USA), 10 ng/mL erythropoietin (EPO) (R&D), and 10^–4^ M β-mercaptoethanol (Sigma, USA).

E14.5 fetal liver cells (FLCs) were mechanically dissociated in PBS containing 10% FBS and then labeled with FITC-conjugated anti-Ter119 antibody (clone TER119). Ter119^−^ cells were purified using the FITC Positive Selection Kit (STEMCELL, Vancouver, BC, Canada) following the manufacturer’s instructions. Purified cells were seeded in fibronectin-coated 24-well plates at a cell density of 3 × 10^5^/mL in IMDM medium supplemented with 15% FBS, 1% detoxified bovine serum albumin (Sigma), 2 mM l-glutamine (Gibco, Grand Island, NY, USA), 200 μg/mL holo-transferrin, 10 μg/mL recombinant human insulin, 10^–4^ M β-mercaptoethanol, and 10 ng/mL EPO. All cells were grown in humidified CO_2_ incubators at 37 °C with 5% CO_2_.

### 4.3. Flow Cytometry Analysis

Single-cell suspensions of murine bone marrow, spleen, fetal liver, or cultured cells were prepared and stained with a fixable viability dye (BD Biosciences, San Jose, CA, USA) for 10 min at 4 °C to exclude dead cells. Subsequently, cells were incubated with fluorochrome-conjugated antibodies specific for cell surface antigens in FACS buffer (PBS containing 2% FBS and 2 mM EDTA) for 30 min at 4 °C. For erythroid progenitor staining, cells were incubated with a lineage antibody cocktail as well as anti-CD55 (clone RIKO-3, Biolegend, San Diego, CA, USA), anti-CD41 (clone MWReg30, Biolegend), anti-CD105 (clone MJ7/18, Biolegend), anti-CD49f (clone GoH3, Biolegend), anti-CD117 (clone 2B8, Biolegend), anti-TER119 (clone TER119, Biolegend), and anti-CD71 (clone RI7217, Biolegend) antibodies. The lineage antibody cocktail included anti-CD11b (clone M1/70, Biolegend), anti-Ly6G (clone 1A8, Biolegend), anti-CD4 (clone GK1.5, Biolegend), anti-CD8a (clone 53-6.7, Biolegend), and anti-B220 (clone RA3-6B2, Biolegend) antibodies. For Ki67 staining, after surface staining, cells were fixed and permeabilized using the BD Fixation/Permeabilization Solution Kit (BD Biosciences) according to the manufacturer’s instructions and then incubated with the Ki67 antibody (clone SolA15, Invitrogen, Carlsbad, CA, USA). Flow cytometry assays were conducted on a BD LSRFortessa X-20 (BD Biosciences) and further analyzed using FlowJo software (version 10) (Tree Star). Detailed information about antibodies is provided in Supplemental Table S1.

### 4.4. Measurement of Protein Synthesis Rate

Protein synthesis rates were measured based on O-propargyl-puromycin (OP-Puro) incorporation. For in vivo assessments, mice were injected intraperitoneally with OP-Puro (50 mg/kg) (MCE, Shanghai, China), while the vehicle group received PBS. One hour after administration, bone marrow or spleen samples were obtained for immunofluorescence or flow cytometry analysis. For ex vivo assessments, cells were incubated with OP-Puro (20 μM) for 1 h at 37 °C prior to harvesting. Following cell surface staining, cells were fixed in 1% paraformaldehyde (PFA) for 15 min at 4 °C and permeabilized with the BD Fixation/Permeabilization Solution Kit for 5 min at room temperature (RT) in the dark. Subsequently, OP-Puro-labeled polypeptides were detected using the Click-iT Plus OPP Alexa Fluor 488 Kit (Thermo Fisher Scientific, Waltham, MA, USA) according to the manufacturer’s protocol.

### 4.5. Immunofluorescence Staining

Tissues were fixed in 4% PFA and dehydrated in 30% sucrose overnight at 4 °C, then embedded in OCT compound (Sakura Finetek, Torrance, CA, USA) and frozen at −80 °C. Sections (20 µm thick) were permeabilized in pre-cooled methanol at −20 °C for 30 min, followed by incubation with blocking buffer (PBS containing 0.3% Tween 20, 1% bovine serum albumin, 1% FBS, and 0.1 M Tris-HCl) for 1 h at RT. Sections were stained with primary antibodies diluted in blocking buffer for 3 h at RT or overnight at 4 °C, and finally incubated with secondary antibodies for 3 h at RT. Fluorescence images were acquired using a Leica SP8 confocal microscope (Leica, Wetzlar, Germany) and analyzed with Imaris (version 9.7.0, Bitplane, Oxford, UK).

### 4.6. eEF2 Phosphorylation Detection

Mouse bone marrow, spleen, or cultured cells were collected and subjected to cell surface staining at 4 °C for 30 min. Cells were then fixed in 1% PFA for 15 min at 4 °C in the dark, followed by washing and permeabilization using the BD Fixation/Permeabilization Solution Kit. For phospho-eEF2 (Thr56) detection, cells were stained with the Phospho-eEF2 (Thr56) antibody (CST, Danvers, MA, USA) diluted in BD Perm/Wash buffer for 30 min at 4 °C. After washing twice with BD Perm/Wash buffer, cells were incubated with a goat anti-rabbit secondary antibody for 30 min at 4 °C. All stained samples were analyzed by flow cytometry.

### 4.7. Western Blot and Quantitative PCR Assay

For western blot, cells were washed with PBS and then lysed by RIPA lysis buffer containing Protease Inhibitor Cocktail (Roche, Basel, Switzerland) and phosphatase inhibitor (Beyotime). Protein concentrations were determined using the BCA Protein Assay Kit (Yeasen, Shanghai, China). Equal amounts of proteins were separated by 8–12% SDS-PAGE and transferred to polyvinylidene fluoride (PVDF) membranes (Merck Millipore, Darmstadt, Germany). Membranes were blocked with 5% non-fat milk in TBST (50 mM Tris, 150 mM NaCl, 1% Tween-20, pH 7.4) for 1 h at RT, followed by incubation with primary antibodies overnight at 4 °C. After washing three times with TBST, membranes were incubated with HRP-conjugated secondary antibodies (CST) diluted 1:5000 in 5% non-fat milk for 1 h at RT.

For qPCR, total RNA was extracted using TRIzol Reagent (Ambion, Austin, TX, USA) following the manufacturer’s instructions and quantified with a NanoDrop spectrophotometer. cDNA was synthesized through reverse transcription using the Hifair II 1st Strand cDNA Synthesis Kit (Yeasen). Subsequent qPCR was performed with SYBR Green Realtime PCR Master Mix (TOYOBO, Osaka, Japan) on a ViiA7 real-time PCR system (Applied Biosystems, Waltham, MA, USA). Relative gene expression levels were calculated based on the 2^–∆∆Ct^ method and normalized to the housekeeping genes *18s* or *Actb*. The primer sequences used for qPCR are listed in Supplemental Table S2.

### 4.8. Proteomic Analysis

Murine bone marrow cells were subjected to cycloheximide (CHX) treatment (0.5 µg/mL; Selleck, Houston, TX, USA) during the final 6 h of a 30 h in vitro culture with EPO and SCF. Then, cells were harvested and stained with surface antibodies against CD71 and Ter119. CD71^+^Ter119^+^ erythroid cells were sorted on a BD FACS Aria III (BD Biosciences) and washed twice with cold PBS prior to being frozen in liquid nitrogen for proteomic analysis, which was performed using a mass spectrometer in data-independent acquisition (DIA) mode. Briefly, cells were lysed in 2% SDS buffer containing 50 mM DTT for 20 min at RT, and the supernatants were boiled at 100 °C for 5 min before alkylation with iodoacetamide for 1 h at RT. Proteins were precipitated with pre-cooled acetone and digested overnight at 37 °C with sequencing-grade modified trypsin (Promega, Madison, WI, USA). Tryptic peptides were obtained by centrifugation at 14,000 g for 20 min at 20 °C, purified using C18 Ziptips, and eluted with 0.1% TFA in 50–70% acetonitrile. The eluted peptides were lyophilized using a SpeedVac (ThermoSavant, Holbrook, NY, USA), and the iRT peptides (Biognosys, Schlieren, Switzerland) were spiked into the sample according to the manufacturer’s instructions prior to analysis. DIA was performed on an Orbitrap Exploris 480 coupled to a FAIMS and EASY-nanoLC 1200 system (Thermo Scientific). The peptides were reconstituted in 0.1% formic acid and separated on a 20 cm analytical column using a 120 min gradient. The DIA data were processed and analyzed by Spectronaut 18 (Biognosys AG) with default settings. The resulting sequences were analyzed using the mouse Uniprot FASTA database.

### 4.9. RNA Sequencing

E14.5 FLCs were purified for Ter119^+^ erythroblasts, followed by RNA extraction as described above. RNA quality was assessed using an Agilent 2100 Bioanalyzer (Agilent Technologies, Santa Clara, CA, USA). mRNAs were enriched with Oligo (dT) beads, and RNA libraries were constructed using the NEBNext Ultra RNA Library Prep Kit for Illumina (New England Biolabs, Ipswich, MA, USA) following the manufacturer’s instructions. Sequencing was conducted on an Illumina Novoseq 6000 by Gene Denovo Biotechnology Co. (Guangzhou, China). Low-quality reads were removed, and the filtered clean reads were mapped to the reference genome using HISAT2 2.1.0. The expression abundance and variations were quantified by calculating TPM (Transcripts per kilobase of exon model per million mapped reads) values using RSEM software (v1.3.3). Differentially expressed genes were analyzed by DESeq2.

### 4.10. Ribosome Profiling

E14.5 FLCs were purified for Ter119^+^ erythroblasts and washed twice with cold PBS containing cycloheximide (100 ug/mL) at 4 °C for 5 min to block translational elongation. Cells were then rapidly frozen in liquid nitrogen for 1 h and stored at −80 °C for ribosome profiling. In brief, cell extracts were prepared in lysis buffer, triturated through a 26-G needle, and centrifuged to collect the supernatant. Ribosome-protected fragments (RPFs) were generated by incubating lysates with RNase I (New England Biolabs) and DNase I (New England Biolabs) for 45 min at RT, followed by inactivation with SUPERase·In RNase inhibitor (Ambion). RPFs with a size larger than 17 nt were isolated using size exclusion columns and the RNA Clean and Concentrator-25 Kit (Zymo Research, Irvine, CA, USA). rRNA was removed by antisense DNA probes and RNase H digestion, with final purification achieved using magnetic beads (Vazyme, Nanjing, China). Subsequently, Ribo-seq libraries were constructed using the NEBNext^®^ Multiple Small RNA Library Prep Set for Illumina^®^ (E7300S, E7300L) and sequenced using Illumina NovaSeq X Plus by Gene Denovo Biotechnology Co. (Guangzhou, China). Low-quality reads were filtered by fastp, and processed RNA-seq reads were aligned to the genome using whole genome alignment by STAR with the 2-pass setting enabled. Gene expression levels were normalized using the TPM (transcripts per kilobase of exon model per million mapped reads) method.

### 4.11. Plasmid Construction and Retroviral Infection of FLCs

To generate retroviral particles, 293T cells were cultured in DMEM medium supplemented with 10% FBS. For overexpression and knockdown of NFE2, the plasmids Migr1-NFE2-IRES-EGFP or MSCV-miR30-shNFE2-EGFP were co-transfected with the retroviral packaging plasmid pCL-Eco at a 3:1 ratio using Lipofectamine™ 3000 (Invitrogen). Viral supernatants were harvested at 48 and 72 h post-transfection, then filtered through a 0.45 μm cell strainer. The sequences of retroviral shRNA oligonucleotides targeting mouse NFE2 are provided in Supplemental Table S2. Retroviral infection of the purified Ter119 negative mouse FLCs was performed as previously described. Briefly, FLCs were resuspended in viral supernatants in the presence of 10 μg/mL polybrene (Sigma) and centrifuged at 800 g for 1.5 h at 32 °C. After spin-infection for 4 h, the viral supernatants were replaced with fresh medium.

### 4.12. Blood Stasis Syndrome Model

Via the tail vein, 10% dextran 500 (molecular weight 500,000) (Yeasen) in 0.9% saline was administered into WT and eEF2_T56M mice at a dosage of 10 mL/kg, and the control group was injected with 0.9% saline. For blood viscosity detection, abdominal aortic blood was collected 1 h after a single dextran 500 injection. For the establishment of the blood stasis mouse model, dextran 500 was injected via the tail vein once daily for four consecutive days, then lung tissues were harvested for hematoxylin and eosin (HE) staining to evaluate the aggregation of red blood cells.

### 4.13. Inferior Vena Cava (IVC) Stenosis Model

Mice were anesthetized with isoflurane, and a median laparotomy was performed to expose the IVC and its lateral branches. Complete ligation of all lateral branches of the IVC was achieved using a 7-0 polypropylene suture. After careful isolation of the IVC from the aorta, a partial ligature (stenosis) of the IVC was conducted by tying a 7-0 polypropylene suture over a blunted 30-gauge needle. The needle was subsequently removed to allow partial restoration of blood flow, and the abdominal cavity was gently closed. Operated mice were euthanized 60–72 h post-surgery, and the thrombi developed within the IVC were obtained for measurement and weighing.

### 4.14. Statistical Analyses

Data are presented as the mean ± standard error of the mean (SEM). All statistical analyses were performed using GraphPad Prism software (v8.0, San Diego, CA, USA). Statistical significance was determined using unpaired two-tailed Student’s *t*-test between two groups and one-way ANOVA with Tukey’s multiple comparison test between multiple groups. Assays were conducted at least three times. *p*-values less than 0.05 were considered significant.

## 5. Conclusions

This study uncovers that erythroid differentiation requires extraordinarily high levels of protein synthesis and is highly sensitive to fluctuations in this process. Concurrently, eEF2 phosphorylation functions as a negative regulatory mechanism to ensure balanced protein synthesis, thereby preventing excessive erythropoiesis. Mechanistic studies demonstrate that eEF2 phosphorylation regulates erythroid differentiation in part by selectively modulating the translation of NFE2. Collectively, these findings highlight the indispensable role of eEF2 phosphorylation in safeguarding erythropoietic homeostasis through the coordination of protein synthesis, shedding light on the intricate translational control underlying erythroid differentiation.

## Data Availability

The raw data supporting the conclusions of this article will be made available by the authors upon request.

## Supplementary Materials

The following supporting information can be downloaded at: https://www.mdpi.com/article/10.3390/ijms26104801/s1.
