# Supplementary material for: A Coordinated Translational Control Mediated by eEF2 Phosphorylation Safeguards Erythroid Differentiation
Source: Int J Mol Sci. 2025 May 16;26(10):4801. doi: 10.3390/ijms26104801 (PMC12112062; doi:10.3390/ijms26104801)
Supplement: Supplementary file 1 [file ijms-26-04801-s001.zip › ijms-3570553-supplementary.pdf]

Supplemental Tables

Supplemental Table S1. Antibodies used in this study

Antibodies	Vendor	Catlog number
BV421 anti-mouse TER-119	Biolegend	Cat# 116234; RRID: AB_10933426
FITC anti-mouse TER-119	Biolegend	Cat# 116206; RRID: AB_313706
PerCP/Cy5.5 anti-mouse TER-119	Biolegend	Cat# 116228; RRID: AB_893636
APC anti-mouse TER-119	Biolegend	Cat# 116212; RRID: AB_313712
BV421 anti-mouse CD71	Biolegend	Cat# 113813; RRID: AB_10899739
FITC anti-mouse CD71	Biolegend	Cat# 113806; RRID: AB_313566
PE anti-mouse CD71	Biolegend	Cat# 113807; RRID: AB_313569
PerCP/Cy5.5 anti-mouse CD71	Biolegend	Cat# 113816; RRID: AB_2565481
APC/Cy7 anti-human/mouse CD49f	Biolegend	Cat# 313627; RRID: AB_2616783
BV510 anti-mouse CD41	Biolegend	Cat# 133923; RRID: AB_2564013
BV785 anti-mouse CD150	Biolegend	Cat# 115937; RRID: AB_2565962
PE anti-mouse CD105	Biolegend	Cat# 120407; RRID: AB_1027699
PE/Cy7 anti-mouse CD55	Biolegend	Cat# 131814; RRID: AB_2800634
BV650 anti-mouse CD117	Biolegend	Cat# 105853; RRID: AB_2876414
BV421 anti-mouse CD4	Biolegend	Cat# 100438; RRID: AB_10900241
APC/Cy7 anti-mouse CD4	Biolegend	Cat# 100414; RRID: AB_312698
BV421 anti-mouse CD8a	Biolegend	Cat# 100753; RRID: AB_10897101
BV421 anti-mouse/human CD11b	Biolegend	Cat# 101235; RRID: AB_10897942
BV650 anti-mouse/human CD11b	Biolegend	Cat# 101259; RRID: AB_11125575
BV421 anti-mouse Ly6G	Biolegend	Cat# 127627; RRID: AB_10897944
AF700 anti-mouse Ly6G	Biolegend	Cat# 127622; RRID: AB_10643269
BV421 anti-mouse/human CD45R/B220	Biolegend	Cat# 103240; RRID: AB_2562905
BV510 anti-mouse/human CD45R/B220	Biolegend	Cat# 103247; RRID: AB_2561394
BV510 anti-mouse CD45	Biolegend	Cat# 103138; RRID: AB_2561392
PE/Cy7 anti-mouse CD45	Biolegend	Cat# 103114; RRID: AB_312978
APC anti-mouse CD38	Biolegend	Cat# 102712; RRID: AB_312932
BV650 anti-mouse IgD	Biolegend	Cat# 405721; RRID: AB_2562731
PE anti-mouse/human GL7	Biolegend	Cat# 144607; RRID: AB_2562925
PE/Cy7 anti-mouse/human GL7	Biolegend	Cat# 144620; RRID: AB_2800676
Alexa Fluor 488 anti-mouse F4/80	Biolegend	Cat# 123120; RRID: AB_893479
FITC anti-Ki67	Invitrogen	Cat# 11-5698-82; RRID: AB_11151330
PE anti-Ki67	Invitrogen	Cat# 12-5698-82; RRID: AB_11150954
Alexa Fluor 647 goat anti-Rabbit	Invitrogen	Cat# A11034; RRID: AB_2576217
Alexa Fluor 488 goat anti-Rabbit	Invitrogen	Cat# A21245; RRID: AB_2535813
Phospho-eEF2 (Thr56) Antibody	CST	Cat# 2331S; RRID:AB_10015204
GATA1 Polyclonal antibody	Proteintech	Cat# 10917-2-AP; RRID: AB_2108279
EEF2 Polyclonal antibody	Proteintech	Cat# 20107-1-AP; RRID: AB_10950401
NFE2 Polyclonal antibody	Proteintech	Cat# 11089-1-AP; RRID: AB_2152928
HBB Polyclonal antibody	Proteintech	Cat# 16216-1-AP; RRID: AB_10598329
ALAS2 Polyclonal antibody	Proteintech	Cat# 30539-1-AP; RRID: AB_3086355
GAPDH Monoclonal antibody	Proteintech	Cat# 60004-1-Ig; RRID: AB_2107436
Beta Actin Recombinant antibody	Proteintech	Cat# 81115-1-RR; RRID: AB_2107436

Supplemental Table S2. Oligonucleotides for qPCR and gene knockdown.

Oligonucleotides	Sequences (5'→3')
qPCR primer: *Nfe2* Forward:	TCTACTCCCCATGTCCCAGA
qPCR primer: *Nfe2* Reverse:	CAGCCTCCATACCCTCTAGC
qPCR primer: *Alas2* Forward:	CCTCAGATGATGGAAGATTTTG
qPCR primer: *Alas2* Reverse:	TCATGAGCTCAAAGTGTACAGGA
qPCR primer: *Gata1* Forward:	TGGGGACCTCAGAACCCTTG
qPCR primer: *Gata1* Reverse:	GGCTGCATTTGGGGAAGTG
qPCR primer: *Hbb* Forward:	TCAGGCTCCTGGGCAATATG
qPCR primer: *Hbb* Reverse:	TTAACCATTGTTCACAGGCAAG
qPCR primer: *Fech* Forward:	TCTTCTTGGACCGAGACCTCATG
qPCR primer: *Fech* Reverse:	TCCAATCCTGCGGTACTGCTCT
qPCR primer: *18S* Forward:	AGTCCCTGCCCTTTGTACACA
qPCR primer: *18S* Reverse:	CGATCCGAGGGCCTCACTA
shRNA sequence: mNfe2-1	CCAGGATTATCCCTCAACTACA
shRNA sequence: mNfe2-2	AAACTACCATACTCCTATGGTA

Supplemental Figures

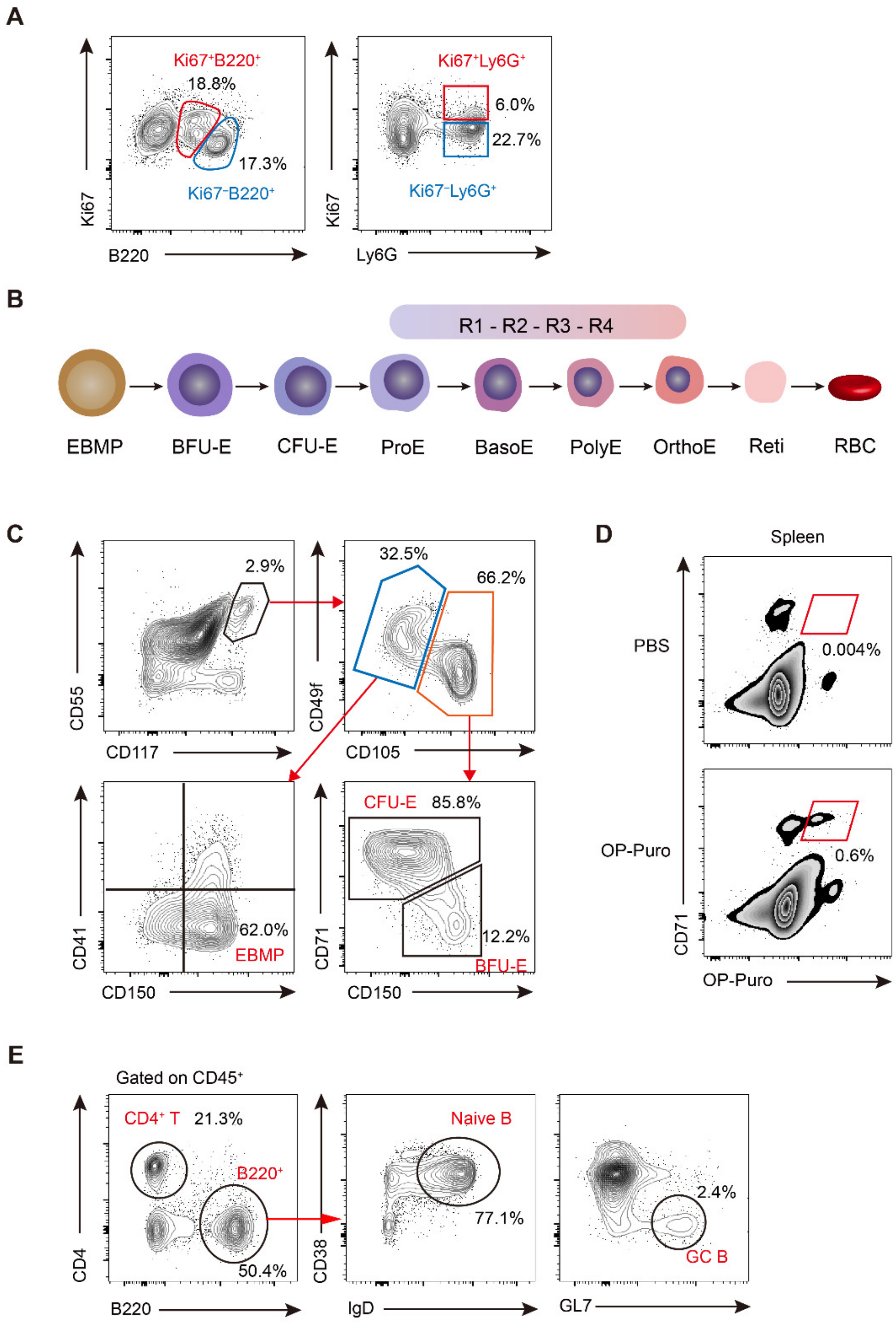

Supplemental Figure S1. Enhanced protein synthesis during erythroid differentiation, related to Figure 1. (A) Representative flow cytometric profiles illustrating the gating strategy for analyzing B cells and neutrophils of Ki67⁺ or Ki67⁻ in bone marrow (BM). **(B)** Schematic diagram depicting the erythroid differentiation trajectory from erythroid-basophil-megakaryocyte-biased progenitors (EBMPs) to mature red blood cells (RBCs). **(C)** Gating strategy for analyzing EBMPs, erythroid burst-forming units (BFU-Es) and erythroid colony-forming units (CFU-

Es) in murine BM. (**D)** Representative flow cytometric profiles of global protein synthesis rates in mouse splenic cells, assessed 1 h after intraperitoneal injection of O-propargyl-puromycin (OP-Puro) at a dose of 25 mg/kg. (**E)** Gating strategy for analyzing $CD4^{+}$T cells, naive B cells ($CD38^{hi}IgD^{+}$) and germinal center (GC) B cells ($CD38^{lo}GL7^{hi}$) in the spleen from sheep red blood cell (SRBC)-immunized mice.

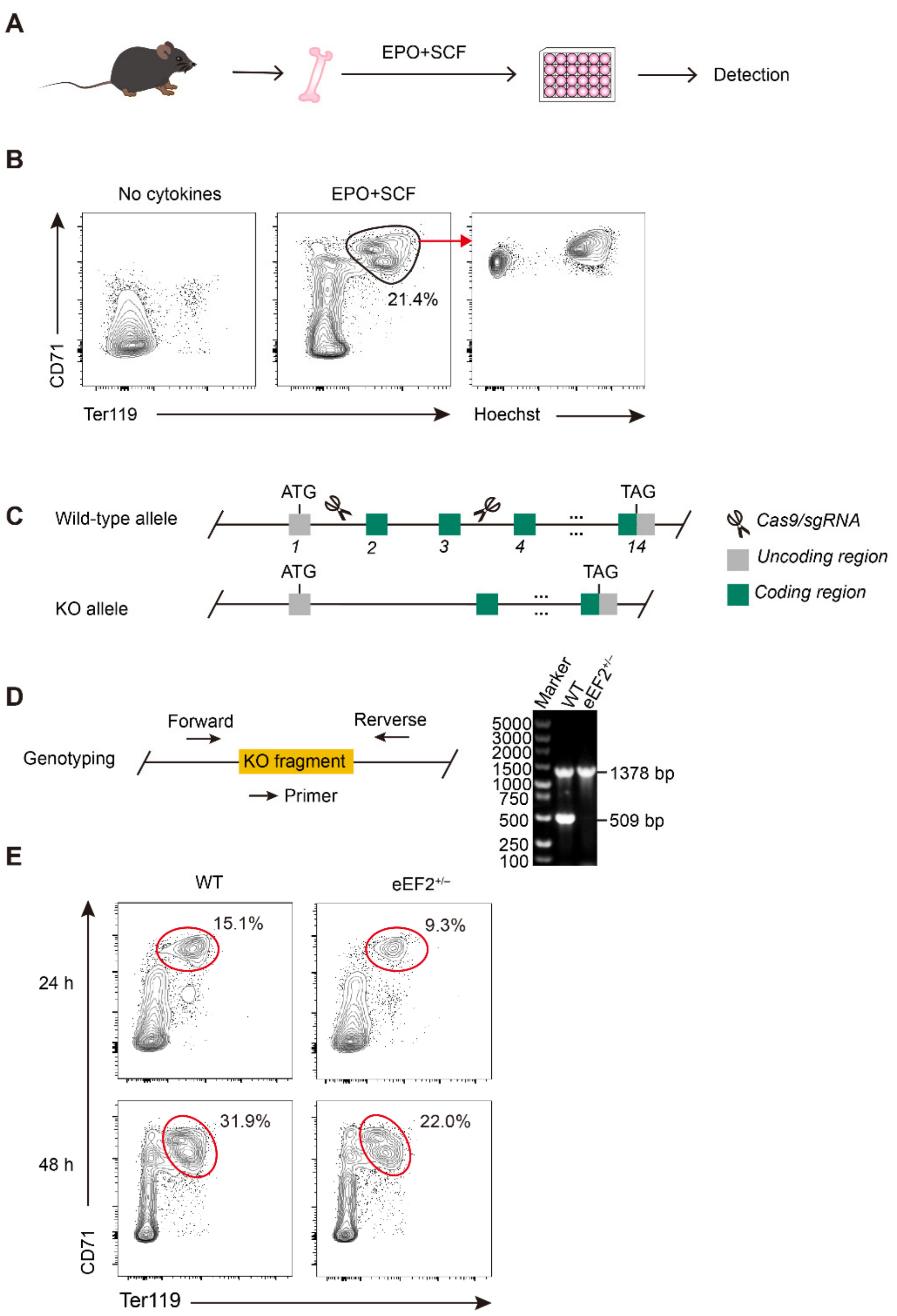

Supplemental Figure S2. Inhibition of protein synthesis constrains erythroid differentiation, related to Figure 2. (A) Experimental scheme. Isolated BM cells were detected after in vitro culture in the presence of erythropoietin (EPO) and stem cell factor (SCF). (**B)** Representative flow cytometric profiles of CD71^{+}Ter119^{+} cells from BM cultured with or without cytokines (EPO and SCF). (**C**) Construction of the eEF2$^{+/-}$ mouse model. (**D)** Genotyping approach (left) for eEF2$^{+/-}$ mice and agarose gel image (right) showing genotyping results of WT and eEF2$^{+/-}$ mice. **(E)** Representative flow cytometric profiles of CD71^{+}Ter119^{+} cells from WT or eEF2$^{+/-}$ BM after in vitro differentiation for 24 or 48 h.

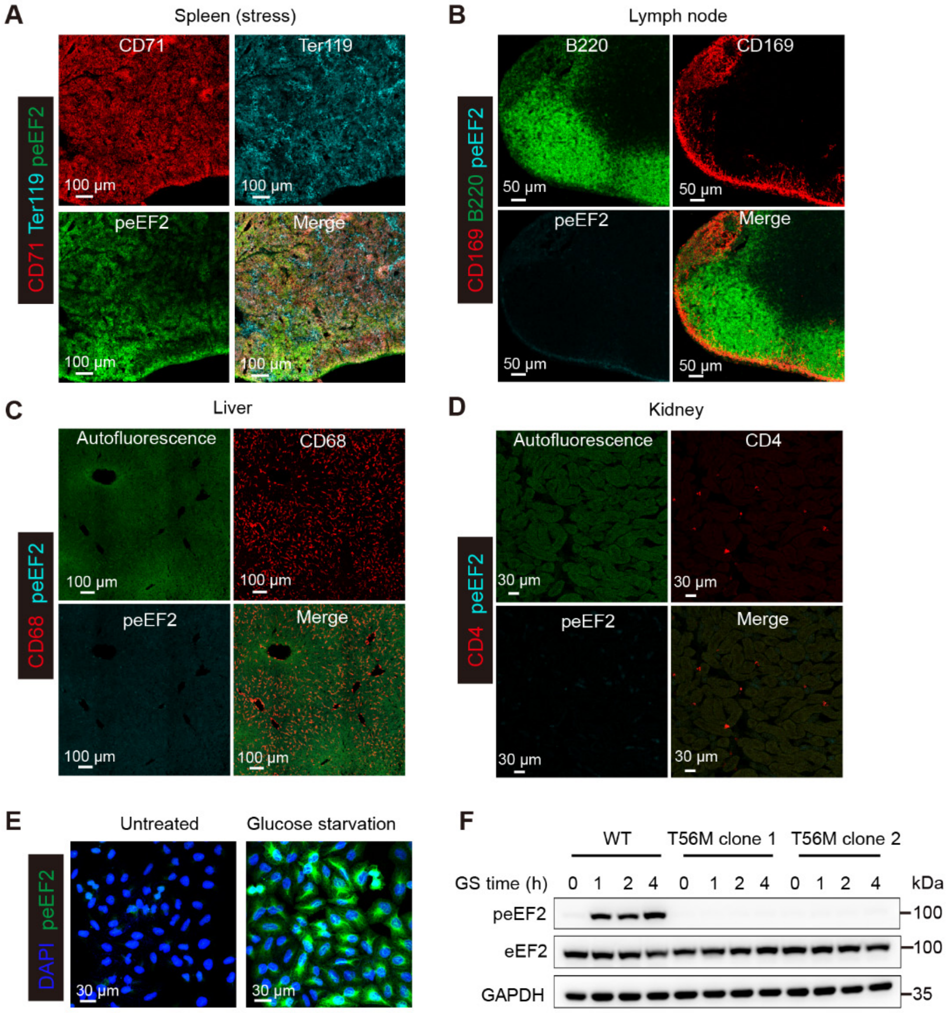

Supplemental Figure S3. eEF2 phosphorylation is tightly associated with erythropoiesis, related to Figure 3. **(A)** Representative confocal images showing immunofluorescence staining of peEF2 (green) in the mouse spleen 4 days post intraperitoneal administration of phenylhydrazine (PHZ; 50 mg/kg) (red: CD71; cyan: Ter119). Scale bars: 100 μm. **(B–D)** Representative confocal images showing immunofluorescence staining of peEF2 (green) in the lymph node (B), liver (C) and kidney (D) from untreated mice. Scale bars: 50 μm (B), 100 μm (C) or 30 μm (D). **(E)** Immunofluorescence staining of peEF2 (green) in HeLa cells with or without glucose starvation (GS) treatment for 1 h (blue: DAPI; green: peEF2). Scale bars: 30 μm. (**F)** Western blot analysis of eEF2 phosphorylation in WT and eEF2_T56M HeLa cells after GS treatment at the indicated time points.

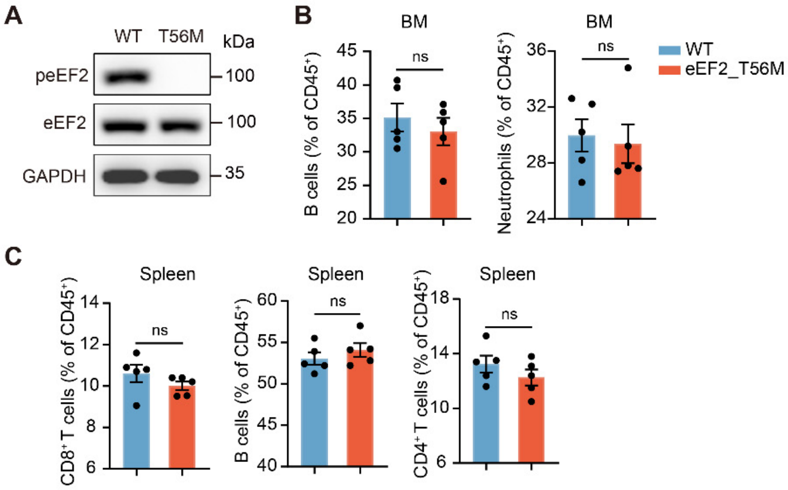

Supplemental Figure S4. Unaltered proportions of other major cell populations in BM and spleen of T56M mice, related to Figure 4. (A) Western blot analysis of peEF2 expression in BM erythroblasts from WT and eEF2_T56M (T56M) mice after in vitro differentiation for 48 h. (**B)** Quantification of the proportions of B cells and neutrophils in BM (*n* = 5). (**C)** Quantification of the proportions of B220$^+$ B cells, CD4$^+$ T cells and CD8$^+$ T cells in the spleen (*n* = 5). Data are presented as mean ± SEM. ns, not significant.

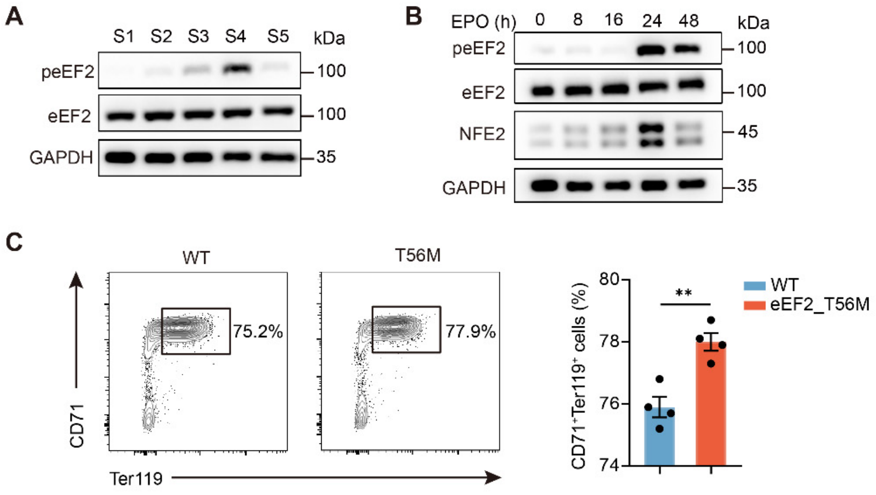

Supplemental Figure S5. Upregulated eEF2 phosphorylation during fetal erythropoiesis, related to Figure 6. **(A)** Western blot analysis of peEF2 expression in S1-S5 subsets of E14.5 mouse fetal liver cells (FLCs). (**B)** Western blot analysis of peEF2 and NFE2 expression in CD71$^+$Ter119$^+$ erythroblasts from FLCs following in vitro differentiation at the indicated time points. (**C**) Representative flow cytometric profiles (left) and quantification of the percentage (right) of CD71$^+$Ter119$^+$ cells from WT or T56M FLCs after 24 h of in vitro differentiation (n = 4). Data are presented as mean ± SEM. **p < 0.01.

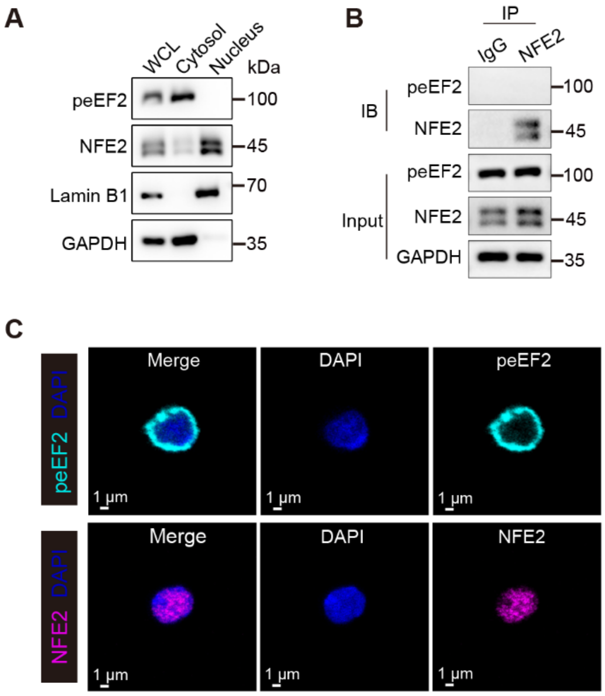

Supplemental Figure S6. No direct binding and spatial localization between peEF2 and NFE2, related to Figure 7. (A) Western blot analysis of total cell lysates, cytoplasmic, and nuclear fractions isolated from murine bone marrow cells after 48 h of in vitro differentiation. (**B)** Immunoprecipitation using an anti-NFE2 antibody on lysates from murine bone marrow cells after 48 h of in vitro differentiation, followed by Western blot analysis to detect peEF2. (**C**) Representative confocal images showing immunofluorescence staining of peEF2 and NFE2 in erythroid cells undergoing in vitro differentiation. (blue: DAPI; cyan: peEF2; magenta: NFE2). Scale bars: 1 μm.

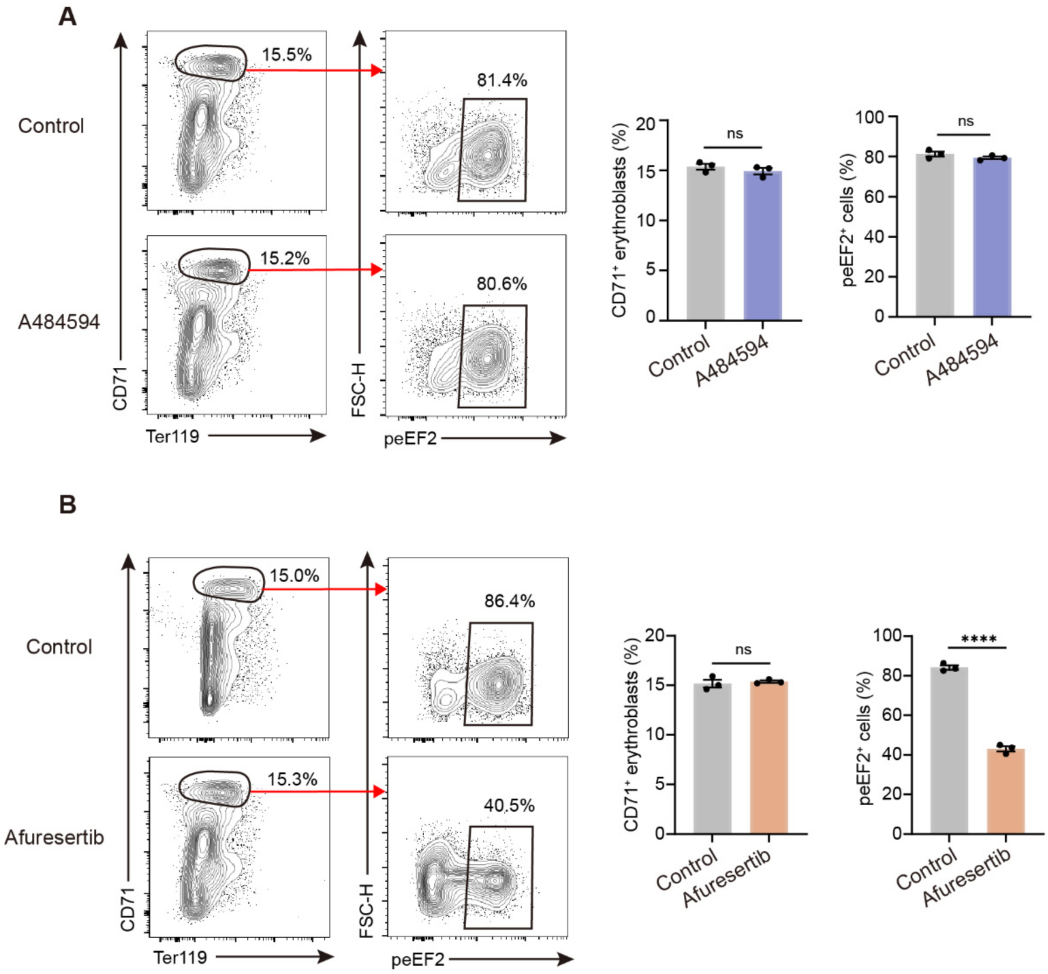

Supplemental Figure S7. eEF2 phosphorylation is independent of eEF2K and potentially regulated by AKT signalling in erythroid cells. (A) Murine BM cells were treated with or without A484594 (an eEF2K inhibitor) for 10 h after 72 h of in vitro differentiation. Representative flow cytometric profiles (top) and quantification of the percentages (bottom) of $CD71^+$ cells and $peEF2^+$ cells ($n = 3$). **(B)** Murine BM cells were treated with or without Afuresertib (GSK2110183) (an AKT inhibitor) for 10 h after 72 h of in vitro differentiation. Representative flow cytometric profiles (top) and quantification of the percentages (bottom) of $CD71^+$ cells and $peEF2^+$ cells ($n = 3$). Data are presented as mean ± SEM. ****$p < 0.0001$; ns, not significant.

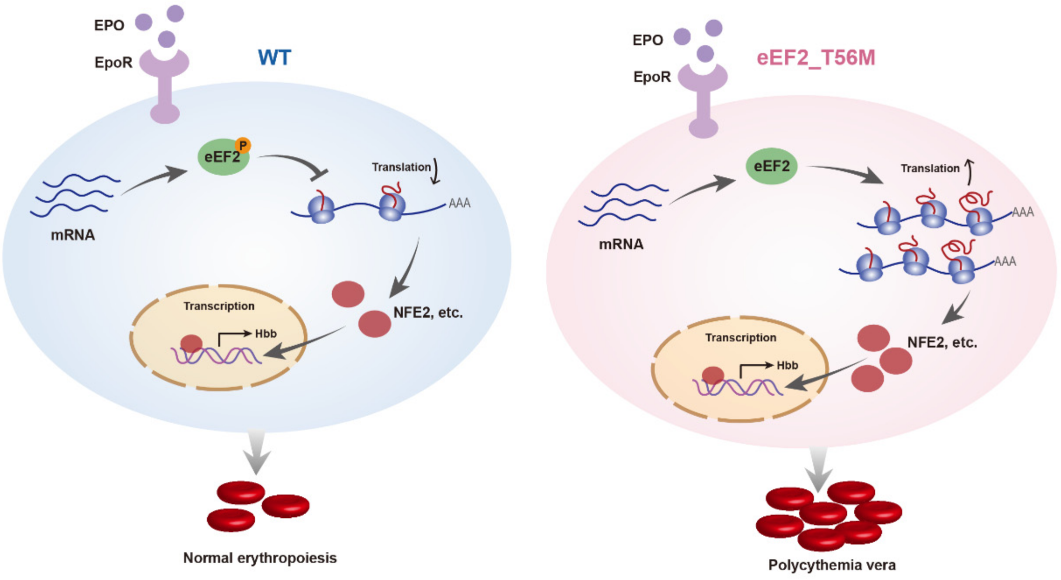

Supplemental Figure S8. Schematic diagram of eEF2 phosphorylation-mediated translational control in erythroid differentiation. eEF2 phosphorylation functions as a molecular brake to maintain erythropoietic homeostasis by coordinating protein synthesis. Disruption of this regulatory mechanism in eEF2_T56M mice results in excessive erythropoiesis.
